# Kyste dermoïde géant de la paroi abdominale: une observation rare

**DOI:** 10.11604/pamj.2015.21.131.7043

**Published:** 2015-06-16

**Authors:** Mbarek Yaka, Abdelkader Ehirchiou, Sifeddine Alkandry, Aziz Zentar

**Affiliations:** 1 Sevice de Chirurgie Viscérale, Hôpital Militaire d'Instruction Mohamed V, Rabat, Maroc

**Keywords:** Kyste dermoïde, géant, paroi abdominale, dermoid cyst, giant, abdominal wall

## Abstract

Les kystes dermoïdes sont des lésions bénignes fréquentes. Ils peuvent être volumineux et entrainer une destruction des tissus avoisinants surtout au niveau du crâne. Ces kystes dermoïdes sont le plus souvent de localisation ovarienne ou testiculaire mais d'autres localisations, principalement axiales, sont décrites. Le kyste dermoïde de la paroi abdominale est rare. Quant aux volumineux kystes, ils sont encore plus rares. La possibilité de leur dégénérescence impose une exérèse chirurgicale. Nous rapportons un cas rare de kyste dermoïde géant de la paroi abdominale antérolatérale, qui peut poser un problème de diagnostic différentiel avec le un kyste hydatique, surtout dans un pays endémique comme le Maroc.

## Introduction

Le kyste dermoïde est une tumeur congénitale bénigne, dont la paroi est constituée d'un épithélium malpighien, pluristratifié, kératinisant et contenant des annexes cutanées. L´atteinte de la paroi abdominale est rare [1, 2], Quant aux volumineux kystes, ils sont encore plus rares et peuvent poser un problème diagnostique avec le kyste hydatique de la paroi abdominale, surtout dans un pays endémique comme le Maroc.

## Patient et observation

**Figure 1:**
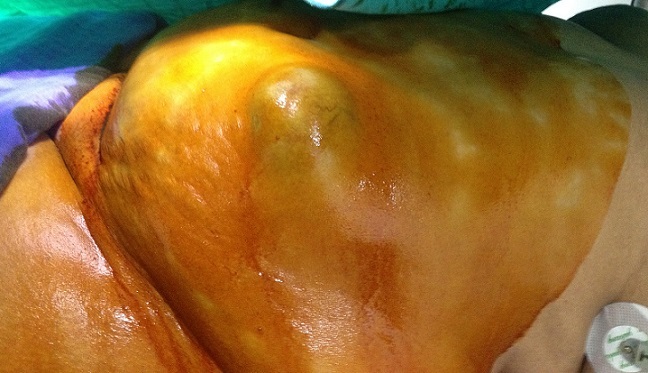
Vue externe: masse de la paroi abdominale anterolatérale gauche

**Figure 2:**
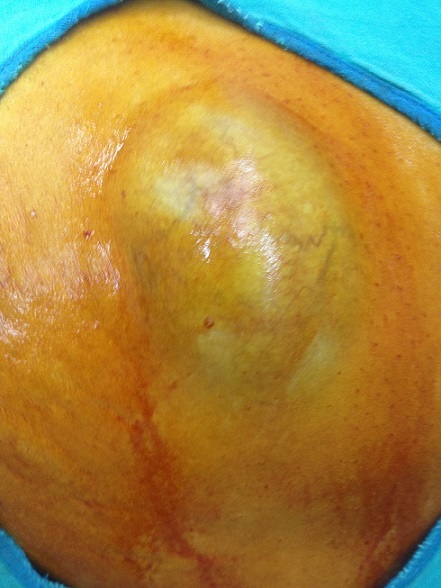
Volumineux kyste dermoide de la paroi abdominal

**Figure 3:**
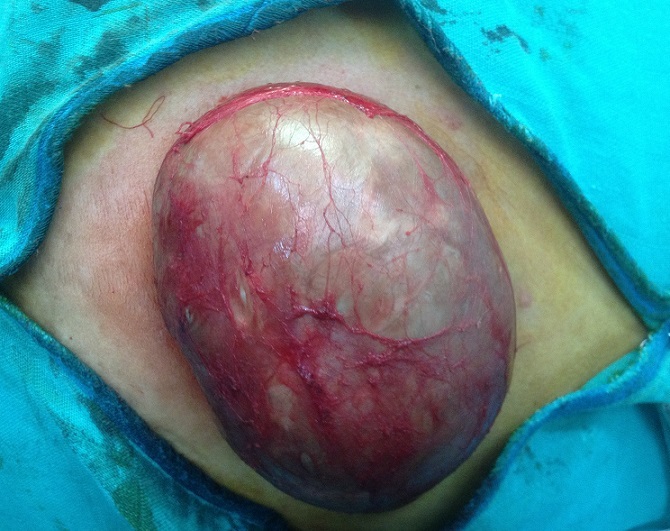
Vue opératoire montrant un kyste dermoide géant de la paroi abdominal

**Figure 4:**
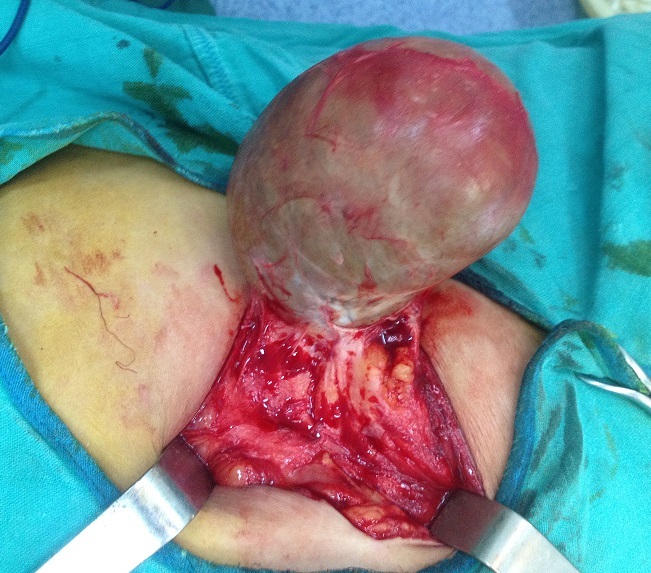
Exérèse complète de kyste dermoide géant de la proi abdominale

Mme A. M. est âgée de 50 ans, ménopausée depuis deux ans, sans antécédents pathologiques particuliers, notamment pas de traumatisme ou de chirurgie pariétale. Le début de la maladie remonte à plusieurs années par l´apparition d´une tuméfaction de la paroi abdominale antérolatérale augmentant progressivement de volume, sans douleur, sans inflammation. L´examen trouve une masse du flanc gauche mesurant 20 cm de grand axe, indolore, mobile par rapport aux plans superficiel et profond. La peau en regard est fine (Figure 1, Figure 2). L'examen des aires ganglionnaires est sans anomalie. L’échographie montre une formation kystique, finement echogène et hyperechogène, la TDM confirmait une masse kystique. La sérologie hydatique (ELISA) est négative. L'exérèse chirurgicale en monobloc de la tumeur est réalisée (Figure 3, Figure 4). L'examen de la pièce montre une tumeur kystique remplie de sébum et de ames de kératine. L’étude histologique, montre une structure bordée par un épithélium malpighien stratifié, kératinisé, et confirme le diagnostic de kyste dermoïde, sans signes de malignité.

## Discussion

Les kystes dermoïdes sont des tumeurs congénitales bénignes d'origine embryonnaire. Dans 10% des cas, ils sont acquis, d'origine traumatique ou iatrogène [1]. Le kyste dermoïde de la paroi abdominale est une tumeur rare, différentes localisations ont été rapporortées. Ils sont axiaux externes fixés à une extrémité ou l'autre du corps (sacrococcyx, encéphale), para-axiaux le plus souvent gonadiques, axiaux internes (selle turcique, médiastin) [2, 3]. Les kystes dermoïdes de l'ovaire sont les plus fréquents et constituent 20% des tumeurs ovariennes de l'adulte, ils sont bilatéraux dans 12% des cas. Par ordre de fréquence croissante, les tératomes extragonadiques ont été médiastinaux antérieurs, rétro péritonéaux, sacro coccygiens [4, 5]. Les kystes dermoïdes sont à contenu graisseux liquidien ou solide. Ils peuvent être la source de complications inflammatoires. Les kystes dermoïdes sont à contenu graisseux liquidien ou solide. Ils peuvent être la source de complications inflammatoires, infectieuses, se rompre dans une cavité et être à l'origine de péritonite chimique ou fistuliser dans un organe creux lors d'une évolution tumorale se rompre dans une cavité et être à l'origine de péritonite chimique ou fistuliser dans un organe creux lors d'une évolution tumorale]. La complication la plus fréquente des kystes dermoïdes a été la dégénérescence tumorale, le plus souvent aux dépens de l’épithélium malpighien en carcinome épidermoïde [1].

Le diagnostic différentiel de kystes dermoïdes doit se faire avec les autres tumeurs kystiques comme les kystes hydatiques. L’élévation de l'antigène carcino-embryonnaire et du squamous cell carcinoma antigen ainsi qu'un kyste dermoïde de diamètre supérieur à 10 cm serait fortement en faveur d'une transformation maligne en particulier pour les kystes dermoïdes ovarien [1, 6].

## Conclusion

Le kyste dermoïde géant de la paroi abdominale est exceptionnel et peut simuler un kyste hydatique. La transformation en carcinome squameux est possible.
